# Labyrinthin Expression Is Associated with Poor Prognosis in Patients with Non-Small-Cell Lung Cancer

**DOI:** 10.3390/cancers15030924

**Published:** 2023-02-01

**Authors:** Weijie Ma, Jie Zeng, Dennis J. Montoya, Kyra Toomey, Chihong Zhou, Shuai Chen, Dingning Liu, Michael Babich, James A. Radosevich, Tianhong Li

**Affiliations:** 1 Division of Hematology/Oncology, Department of Internal Medicine, University of California Davis School of Medicine, University of California Davis Comprehensive Cancer Center, Sacramento, CA 95817, USA; 2 Department of Pathology and Laboratory Medicine, Dartmouth Hitchcock Medical Center, Geisel School of Medicine at Dartmouth, Lebanon, NH 03755, USA; 3 Medical Service, Hematology and Oncology, Veterans Affairs Northern California Health Care System, 10535 Hospital Way, Mather, CA 95655, USA; 4 Department of Biochemistry and Molecular Medicine, University of California Davis, Sacramento, CA 95817, USA; 5 Department of Pathology and Laboratory Medicine, University of California Davis School of Medicine, Sacramento, CA 95817, USA; 6 Division of Biostatistics, Department of Public Health Sciences, University of California Davis, Davis, CA 95616, USA; 7 Department of Statistics, University of California Davis, Davis, CA 95616, USA; 8 LabyRx Immuno-Oncology, Sacramento, CA 95817, USA

**Keywords:** labyrinthin, lung adenocarcinoma, lung squamous cell carcinoma, non-small-cell lung cancer, RNA sequencing, prognosis, TCGA, tissue microarray

## Abstract

**Simple Summary:**

Adenocarcinomas represent nearly 40% of all cancer types, and account for more than 70% of cancer-related deaths. Labyrinthin (LAB) is a novel cancer neoantigen expressed on the surface of adenocarcinoma cells of various cancer types. Several treatment strategies are being developed for targeting LAB as a pan-adenocarcinoma target. This study aimed to develop an immunohistochemistry (IHC) assay to detect LAB expression on archival tumor specimens of non-small-cell lung cancer (NSCLC) and characterize the clinical significance of LAB expression. We also explored LAB mRNA expression by RNA sequencing using NSCLC data in The Cancer Genome Atlas (TCGA), and correlated with clinical phenotype and outcome. Further study is warranted to validate LAB IHC and LAB RNA expression as independent biomarkers for selecting patients for cancer therapy targeting LAB.

**Abstract:**

To determine Labyrinthin (LAB) expression in non-small-cell lung cancer (NSCLC), we immunostained and scored for LAB immunohistochemistry (IHC) expression on sections of tissue microarrays (TMAs) prepared from 256 archival tissue blocks of NSCLC. Propensity-score-weighted Kaplan–Meier curves and weighted Cox models were used to associate LAB expression with overall survival. LAB mRNA expression was assessed in The Cancer Genome Atlas (TCGA) and correlated with clinical phenotype and outcome. Positive LAB IHC expression (>5% of tumor cells) was detected in 208/256 (81.3%) of NSCLC samples, and found in both lung adenocarcinomas (LUAD) and lung squamous cell cancer (LUSC). LAB positivity was associated with poor overall survival (HR = 3.56, 95% CI: 2.3–5.4; *p* < 0.0001) and high tumor differentiation grade or metastasis compared with negative LAB expression. Univariant and multivariate survival analyses demonstrated LAB expression as an independent prognostic factor for NSCLC patients. LAB RNA expression in TCGA-LUAD was higher in primary and advanced-stage tumors than in normal tissue, and was associated with poorer overall survival. No significant differences or associations were found with LAB RNA expression in TCGA-LUSC. The LAB IHC assay is being used to identify candidate cancer patients for the *first-in-human* phase I trial evaluating the LAB vaccines (UCDCC#296, NCT051013560).

## 1. Introduction

Throughout the history of cancer care, adenocarcinomas have continued to be the most common histologic type of solid tumors, mainly due to their ability to arise from nearly anywhere in the body. Labyrinthin (LAB) is a distinct pan-adenocarcinoma tumor-specific target composed of 255 amino acid (AA) proteins within the 758 AA of the intracellular aspartyl/asparaginyl beta-hydroxylase (ASPH) that was discovered in 1985 [1]. ASPH has diverse molecular functions [2]. The ASPH protein aggregates on the surface of tumor cells, and contributes to inducing tumor cell migration, infiltration, metastasis, and immune modulation. ASPH upregulation activates the Notch, MAPK and PI3K signaling pathways, delays tumor cell senescence, destroys the integrity of mitochondria, and subsequently leads to tumor development [2]. Compared with the majority of ASPH subtypes, LAB does not have the endoplasmic reticulum (ER) anchoring sequence [1]. LAB is tightly associated with the plasma membrane and solely expressed on the extracellular surface of human adenocarcinoma cells, regardless of tissue origin [3]. An increasing body of evidence suggests that LAB is a functional cell-surface marker and a promising cancer neoantigen [1]. Overexpression of LAB has been detected in multiple adenocarcinoma types, including lung, breast, and colon. Human WI-38 lung fibroblast cells transfected with LAB sense cDNA displayed a cancerous phenotype. Human A549 lung adenocarcinoma cells have high LAB expression; antisense transfection made A549 cells appear more normal. LAB mRNA and protein are increased in tumor cells under varying physiological calcium concentrations. Furthermore, various external calcium levels and intracellular pH changes (which alter intracellular free calcium levels) also modulated LAB expression in cancer cells [4,5]. The mouse monoclonal antibody (MCA) 44-3A6 was developed against LAB from A549 cells, which specifically recognized the membrane-associated LAB protein (40 kilodalton) [6]. MCA 44-3A6 inhibited the phosphorylation and proliferation of A549 and tumor growth in preclinical tumor models of various adenocarcinomas [6,7]. Furthermore, MCA 44-3A6 detected LAB expression on both fresh tumor specimens and formalin-fixed, paraffin-embedded (FFPE) tumor specimens [6,7]. Epitope mapping revealed MCA 44-3A6 bound to an epitope of six amino acids (117–122; “P” peptide) in LAB, which was highly immunogenic. Further mechanistic studies are needed to confirm the molecular functions of LAB in carcinogenesis and tumor progression.

LAB is being evaluated as a novel cancer neoantigen for peptide vaccines in patients with advanced adenocarcinomas (UCDCC#296, NCT051013560) [8]. However, there is no biomarker assay for selecting candidate patients for the LAB vaccine study, and no study characterizing the prognosis of LAB expression using archival formalin-fixed, paraffin-embedded (FFPE) clinical specimens. Non-small-cell lung cancer (NSCLC) accounts for the majority of lung cancer, which is the leading cause of cancer-related deaths worldwide [9]. Despite advancements in early detection and treatment, the prognosis for the majority of NSCLC patients remains poor [10,11]. Novel therapeutic targets and treatment strategies are needed to improve the survival of patients with NSCLC [12,13,14,15]. 

This study aimed to develop an immunohistochemistry (IHC) assay to identify patients with high LAB expression on archival FFPE clinical specimens of NSCLC and confirm the association of high LAB expression with clinical outcome. Considering that commonly used antibodies can recognize epitopes in both ASPH and LAB, the clinical significance of an assay developed to distinguish LAB RNA expression was validated in a publicly assessable database of The Cancer Genome Atlas (TCGA). 

## 2. Materials and Methods 

### 2.1. Tissue Microarrays (TMAs)

Tissues were obtained via an exempted Institutional Review Board protocol (UC Davis Cancer Center Biorepository, IRB 293828,) with deidentified patient demographic information, pathology, and survival outcomes. TMAs were prepared as described previously [16]. Briefly, cylindrical cores with a diameter of 0.6 mm and length of 0.4 mm were cut from FFPE tissue blocks of NSCLC tissue and three cores of tumor or normal adjacent control were placed together in the same TMA block. Three cores of normal liver and kidney tissues were also included as normal controls. One core was stained with hematoxylin and eosin (H & E) for general histological analysis.

### 2.2. Immunohistochemistry (IHC) Stain

Slides were prepared by first cutting FFPE tissue sections (4-μm) from each TMA. Slides were then deparaffinized and antigen retrieval performed with heat-induced-epitope-retrieval solution (pH 9) (DAKO, Agilent, Santa Clara, CA, USA). Subsequently, slides were incubated in dual endogenous enzyme block (DEEB) blocking solution in a DAKO Autostainer. TMA slides were immunolabeled by IHC for LAB expression using LAB antibody MCA 44-3A6. MCA 44-3A6 is a mouse monoclonal anti-labyrinthin antibody that was produced using the standard hybridoma technology (HB-8986^TM^, ATCC, Manassas, VA, USA) [3,4]. The slides were incubated with the hybridoma supernatant containing MCA 44-3A6 for 60 min, washed with PBS, then visualized by EDL solution (DAKO EnVision and Dual Link System-HRP). All slides were counterstained with hematoxylin. IHC slides were scored by two blinded readers (WM and JZ) and verified by two pathologists (WM and CZ) blinded to clinicopathologic information. LAB protein expression was quantified using the percentage of positive cells. A scale of increasing LAB expression was defined as 0 (0–5%), 1+ (6–20%), 2+ (21–50%), and 3+ (51–100%). A tumor proportion score (TPS) was also calculated as the percentage of viable tumor cells showing partial or complete membrane staining at any intensity. The specimen was considered to have positive LAB expression if TPS > 5% and negative LAB expression if TPS ≤ 5%.

### 2.3. Statistical Analysis for IHC Data

All baseline demographics and patient characteristics were compared between different LAB expression level groups by Fisher’s exact tests, except for the continuous variable, age, in which a Kruskal–Wallis Test was utilized. Multivariate logistic regression with backward-variable section was applied to patient and tumor characteristics to examine factors associated with positive LAB expression (TPS > 5%) versus negative LAB expression (TPS ≤ 5%). The Kaplan–Meier plots and log-rank test were employed to compare overall survival (OS) between different LAB expression levels. Univariate survival analysis and multivariate analysis were performed with Cox proportional hazards models using OS as outcome. Schoenfeld residuals were examined to ensure that the proportional hazards assumption holds. To account for potential confounding and covariate imbalances between different LAB expression groups, we used propensity score analysis as previously described [16]. This method has been shown to produce less-biased treatment effect estimates for survival outcomes than stratification or covariate adjustment based on the propensity score [17]. Thus, we used a propensity-score-weighted Kaplan–Meier estimator, using the inverse of the propensity score [18]. Significant covariates in univariate and multivariate survival analyses were used in the propensity score model using logistic regression. We also performed subgroup analyses for different patient characteristics groups. As there was a high proportion of unknown metastasis status (63% of all patients) in the database, we performed additional sensitivity analyses using the multiple imputation method for unknown metastasis based on missing at random (MAR) assumption [19] and further including metastasis status in multivariate analysis. All analyses were performed using SAS 9.4 (SAS Institute Inc., Cary, NC, USA) and R 4.0.4 (R Foundation for Statistical Computing, Vienna, Austria). All *p*-values were two-sided, and a *p* ≤ 0.05 was considered statistically significant. 

### 2.4. LAB RNA Expression Analysis

Exon-level RNA expression and clinical data were retrieved from the TCGA splicing variants database (TSVdb) [20], accessed on 11 October 2022. In total, 515 lung adenocarcinoma (LUAD) tumors, 59 LUAD adjacent-normal tissues, 501 lung squamous cell carcinoma (LUSC) tumors, and 51 LUSC adjacent-normal tissues were included [21]. LAB-specific exons were found in the following ASPH *loci* (chr8:62537095-62566219, 62593523-62593595, 62596598-62596747) and averaged to estimate the LAB RNA expression values. The average LAB exon expression was then utilized to separate the cohorts into LAB-high and LAB-low expression groups, on which log-rank tests for significant difference (*p* < 0.05) were conducted for the survival analysis. Survival data plots were produced using the *survminer* package of R software, version 0.4.9 (R Foundation for Statistical Computing, Vienna, Austria) [22] and Graph Prism software, version 8.21 (Dotmatics, Boston, MA, USA). High LAB expression was defined as primary tumor samples with higher than the median LAB expression across all tumor samples. 

## 3. Results

### 3.1. Patient Characteristics and LAB Expression in NSCLC

**Figure 1:**
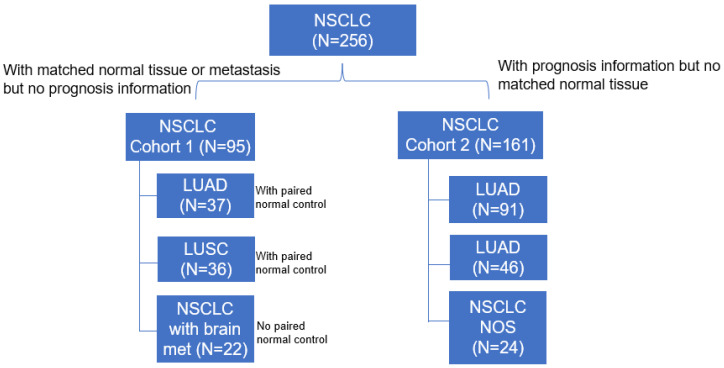
Study flow chart. The distribution of lung cancer types of TMA is illustrated. Abbreviations: NSCLC, non-small-cell lung cancer; LUAD, lung adenocarcinoma; LUSC, lung squamous cell carcinoma; NOS, not other specified.

Figure 1 summarizes all the 256 NSCLC cases included in this study. Cohort 1 contained TMA samples from 95 NSCLC patients, which included 37 LUAD with matched non-cancerous controls, 36 LUSC with matched non-cancerous controls, and 22 NSCLC cases with brain metastases. Cohort 2 had tumor tissue samples from 161 NSCLC patients, which included 91 LUAD, 46 LUSC, and 24 cases with unspecified histology types. The patient demographics and tumor characteristics for cohort 1 and cohort 2 are summarized in Table S1 and Table S2, respectively.

**Figure 2:**
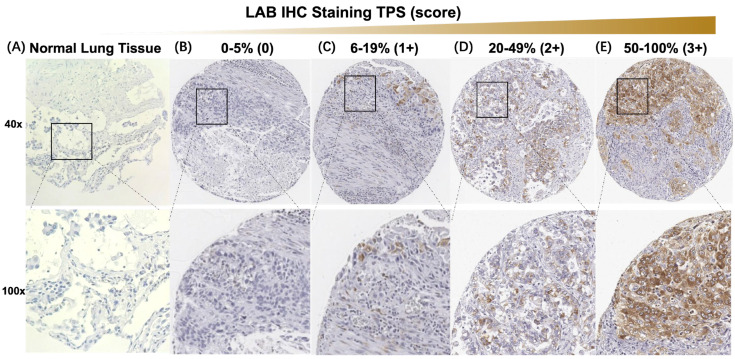
Atlas of LAB expression by IHC. (**A**) Representative pictures of LAB stain in normal lung tissue. (**B**–**E**) Atlas of representative pictures of 0, 1, 2, and 3 IHC scores for LAB expression in NSCLC TMAs. Abbreviations: LAB, labyrinthin; IHC, immunohistochemistry; NSCLC, non-small-cell lung cancer; TMA, tissue microarray; TPS, tumor proportion score.

**Figure 3:**
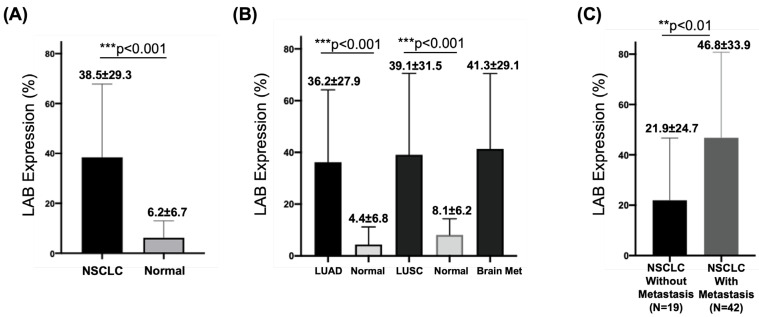
LAB expression and NSCLC metastasis. (**A**) Increased LAB expression was detected in NSCLC compared to matched, non-cancerous tissues. (**B**) Increased LAB expression was detected in LUAD and LUSC compared to matched, non-cancerous tissues, and brain metastasis. (**C**) Higher LAB expression was associated with NSCLC metastasis. ** *p* < 0.01 and *** *p* < 0.001 for statistical significance.

Figure 2 illustrates the atlas of LAB expression by IHC. Compared to fresh tumor specimens that have LAB expression on the cell surface, LAB expression was detected in both cytoplastic and surface compartments due to the cell permeability of surface protein during fixation. LAB expression was detected in 83 (87.4%) of 95 NSCLC specimens in cohort 1, which included weak (1+) in 16 (16.8%) cases, moderate (2+) in 35 (36.8%) cases, and strong (3+) in 32 (33.7%) cases. LAB had higher median expression in all NSCLC tumors compared to matched non-cancerous normal tissues (38.5 ± 29.3% vs. 6.2 ± 6.7%, *p* < 0.001) (Figure 3A), and LUAD (36.2 ± 27.9% vs. 4.4 ± 6.8%, *p* < 0.001) and LUSC (39.1 ± 31.5% vs. 8.1 ± 6.2%, *p* < 0.001) in cohort 1, respectively (Figure 3B). Furthermore, LAB expression was significantly higher in NSCLC tumors with metastasis compared to those NSCLC tumors without metastasis from patients with metastatic tumors (46.8 ± 33.9% vs. 21.9 ± 24.7%, *p* < 0.01) in cohort 2 (Figure 3C). Despite 62% of cases with unknown metastasis status, the confidence interval of hazard ration (HR) was wide.

### 3.2. High LAB Expression Was Associated with Poor Prognosis in NSCLC

**Figure 4:**
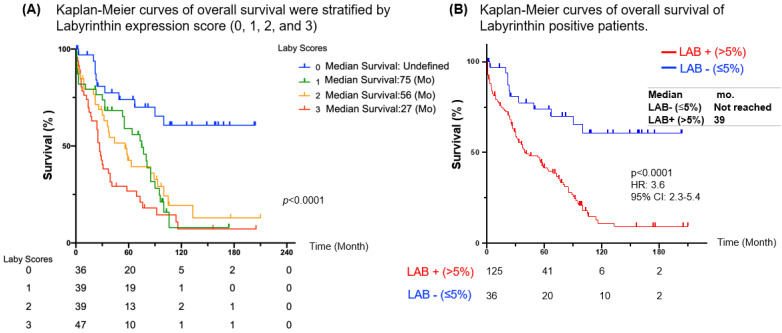
High LAB expression was associated with poor prognosis in NSCLC patients. (**A**) Kaplan–Meier curves of overall survival were stratified by LAB expression score (0, 1, 2, and 3). (**B**) Kaplan–Meier curves of overall survival were stratified by LAB expression (negative vs. positive).

We next investigated the prognosis of LAB expression using NSCLC cases in cohort 2. We found that NSCLC patients with LAB expression (IHC 1+ to 3+) had poor prognosis compared to those NSCLC patients with no LAB expression (IHC 0) (*p* < 0.001). However, there were no significant differences between weak (1+), moderate (2+), and high (3+) LAB expression (Figure 4A). Based on these data, study pathologists recommended to define positive LAB expression as TPS > 5% and negative LAB expression as TPS ≤ 5%. According to this IHC scoring, patients whose NSCLC tumors expressed LAB (TPS > 5% IHC) had poorer prognosis compared with those NSCLC patients with no LAB expression (TPS ≤ 5% IHC) (not reached vs. 39 months, HR: 3.6, 95% CI: 2.3–5.4, *p* < 0.0001) (Figure 4B). The clinicopathological parameters of cohort 2 NSCLC patients with various LAB expression are summarized in Table S3. Of note, the high LAB expression group had a significantly higher proportion of high-grade tumors and metastasis. Univariate and multivariate analyses confirmed that high LAB expression was the strongest independent indicator of unfavorable OS in NSCLC patients (HR: 3.55, 95% CI: 2.30–5.40, *p* < 0.0001; versus HR: 5.9, 95% CI: 2.61–13.34, *p* < 0.0001) (Table 1). Advanced age (≥65 years old) was a weak prognostic factor in both univariant and multivariant analyses (HR: 1.55 and 1.64, *p* = 0.02 and 0.028, respectively) (Table 1). We also performed propensity-score-weighted survival analyses between different LAB expression levels for different patient characteristics groups using Cox proportional hazards models (Table S4). We found that association between LAB IHC expression (TPS > 5%, >20%, and >51%) and poor prognosis was significant in subgroups of NSCLC patients (Table S5). Sensitivity analyses using multiple imputation demonstrated similar conclusions for multivariate analysis when further adjusting for metastasis (Table S6). 

### 3.3. LAB Expression in Histology Subgroups of NSCLC

**Figure 5:**
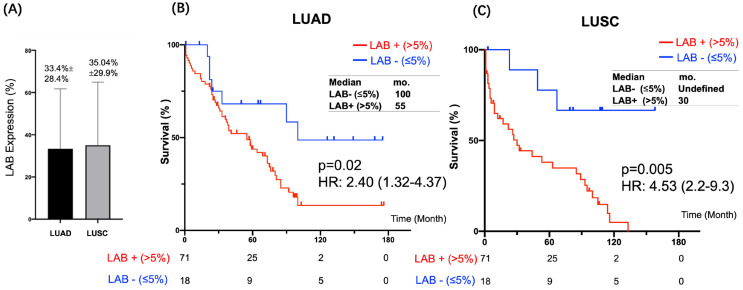
LAB expression in histology subtypes of NSCLC. (**A**) The median LAB expression was higher in LUSC than LUAD. Kaplan–Meier curves of overall survival were stratified by LAB expression (negative vs. positive) in LUAD (**B**) and LUSC (**C**), respectively.

We found the mean score of LAB IHC was higher in LUSC than LUAD in cohort 2 (35.04% ± 29.9% vs. 33.4% ± 28.4%) (Figure 5A), which is consistent with cohort 1 (Figure 3B). Of note, LUSC had high LAB expression in the cytoplasm than surface, while the majority of LUAD had LAB expression on the cell surface. Representative LAB IHC stains in LUAD and LUSC specimens are shown in Figure S1. High LAB IHC score was associated with a statistically significant difference in both LUAD (HR: 2.40, 95% CI: 1.32–4.37, *p* = 0.005) (Figure 5B) and LUSC (HR: 4.53, 95% CI: 2.2–9.3, *p* = 0.005) (Figure 5C), respectively. After adjusting for confounders, propensity-score-weighted survival analysis showed that the association of LAB IHC expression (weak to strong) and poor prognosis was statistically significant in both LUSC and LUAD patients. 

### 3.4. High LAB RNA Expression Was Associated with Poor Prognosis in Patients with LUAD

**Figure 6:**
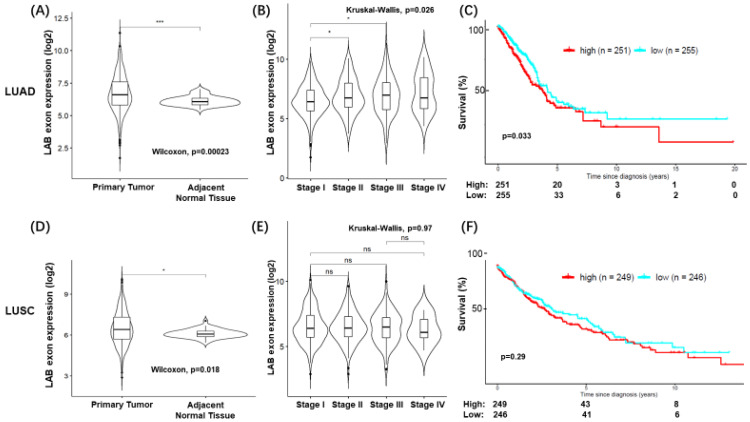
LAB RNA expression in TCGA. Average LAB exon RNA expression was assessed in the TCGA LUAD. Violin plots of average LAB expression in LUAD by tissue type (**A**) or cancer stage (**B**). Pairwise statistical differences assessed by Wilcoxon rank sum test (Mann–Whitney U test) and one-way ANOVA by Kruskal–Wallis tests. (**C**) Kaplan–Meier curves depicting overall survival or patients with higher median LAB RNA expression. Number at risk table shown below. Log-rank test used to evaluate significance. Same analyses applied to TCGA LUSC (**D**–**F**). * *p* < 0.05 and *** *p* < 0.001 for statistical significance. ns for no statistical significance.

We analyzed the RNA expression of LAB using the exon-level expression data from the TCGA-LUAD (*n* = 576) and TCGA-LUSC (*n* = 552) cohorts. Given that LAB is expressed within the ASPH gene, we utilized exon-level expression data obtained from the TCGA splicing variants database (TSVdb) [20]. First, we compared LAB RNA expression between primary tumors and normal tissues. LAB RNA expression was significantly higher in primary tumors for both TCGA-LUAD and TCGA-LUSC, but a higher significance was found in TCGA-LUAD (*p* = 0.00023) vs. TCGA-LUSC (*p* = 0.018) (Figure 6A). Second, we saw an increase in LAB expression in tumors with stage II or stage III disease compared to those with stage I disease in TCGA-LUAD but not TCGA-LUSC (Figure 6B). Similar to the findings in the IHC, high LAB RNA expression was associated with a statistically significant poor prognosis in TCGA-LUAD (*p* < 0.05) (Figure 6C). High LAB RNA expression in TCGA- LUSC trended towards a poorer prognosis but did not reach significance (*p* = 0.29). Thus, LAB expression was associated with poor prognosis in TCGA-LUAD but not TCGA-LUSD in this large and independent TCGA dataset. 

## 4. Discussion

Neoantigens arise from about 10% of the non-synonymous somatic mutations in cancer cells [23]. These neoantigens are important targets for cancer immunotherapy, especially for those patients who progressed on standard of care chemotherapy, targeted therapy, and immune checkpoint inhibitor therapy [24,25,26]. Labyrinthin (LAB) is a promising cancer neoantigen for pan-adenocarcinomas. The mouse monoclonal antibody MCA 44-3A6 detects LAB on FFPE tumor tissues. In the present study, we defined LAB expression as TPS > 5% by IHC in NSCLC specimens. LAB was expressed in the majority (81.3%) of 256 NSCLC specimens by IHC (Figure 2, Figure 3 and Figure 4). NSCLC patients with LAB expression had poorer prognosis compared to those patients with no LAB expression (HR 3.6, *p* < 0.0001) (Figure 4). Compared to the matched, non-cancerous tissues (<8.1%), high LAB IHC expression was found in both LUAD (36.2 ± 27.9%) and LUSC (39.1 ± 31.5%). LAB IHC expression was associated with shorter overall survival compared with those patients with negative LAB expression (HR = 3.56, 95% CI: 2.3–5.4; *p* < 0.0001). Patients with high LAB expression had a significantly higher proportion of high-grade tumors and metastasis (Figure 3). In both univariant and multivariate survival analyses, LAB IHC expression was an independent prognostic factor for NSCLC patients (Table 1). Advanced age (≥65 years old) was also a prognostic factor in both univariant and multivariant analyses.

We detected LAB expression by IHC in both LUAD and LUSC (Figure 5). High LAB expression in LUAD was associated with poor prognosis and shorter overall survival. However, the detection of LAB in LUSC was not expected, as LAB was identified as a pan-adenocarcinoma marker. This was due to the permeability of the membrane, either making LAB internalized into the cytoplasm of tumor cells, or the positive signals reflecting MCA 44-3A6 binding to a shared epitope of intracellular ASPH as previously described. In support, IHC studies that used an entirely different antibody (FB-50) that also cross-recognizes LAB and ASPH found a 10% positive signal in LUSC [27]. The results underscore the importance of sufficient antibody titration in IHC methods in order to increase the ratio of signal-to-noise (i.e., LAB_ext_ ASPH_int_). Thus, it was necessary to develop the RNA assay to distinguish LAB from ASPH.

With the advances in next-generation sequencing technology and computational bioinformatics, we were able to efficiently identify genomic alterations, putative neoantigens, and gene expression profiling in individual tumors for personal oncology in a rapid and cost-effective way. We therefore determined LAB expression using RNA sequencing data from TCGA and prognostic value of LAB RNA expression in LUAD and LUSC (Figure 6). Similar to the LAB IHC results, we found statistically significant high LAB RNA expression in both TCGA-LUAD (*p* = 0.00023) vs. TCGA-LUSC (*p* = 0.018) (Figure 6A) compared to normal tissues. In contrast, there was a positive association between LAB RNA expression and the tumor stage of TCGA-LUAD but not TCGA-LUSC. High LAB RNA expression was also associated with poor prognosis in TCGA-LUAD but not TCGA-LUSC. Thus, LAB remains a cancer biomarker and drug target in LUAD.

Our study has several translational potentials. First, LAB expression can be used to diagnose LUAD, given it is expressed in the >80% of LUAD. Second, this LAB IHC assay can be used to assess LAB expression using archived FFPE tissue specimens for candidate patients for LAB-targeted therapy. However, the present IHC approach could not differentiate surface and cytoplasmic stains. Third, LAB RNA expression can be assessed in RNA sequencing data of clinical specimens. Thus, no additional biomarker assay is needed. Further study is warranted to determine the concordance of LAB IHC and RNA expression in LUAD and adenocarcinomas of other cancer types, as well as clear differentiation between ASPH and LAB-specific RNA expression. This will contribute to their clinical utility. Mechanistic studies are also needed to delineate how LAB mediates the malignant processes and to develop therapeutic strategies for targeting LAB in pan-adenocarcinomas.

## 5. Conclusions

In summary, LAB expression by IHC was associated with poor prognosis in patients with NSCLC. This IHC assay is being used to identify candidate cancer patients for the *first-in-human* phase I trial (NCT05101356) evaluating the LAB vaccine. Further biomarker studies by RNA sequencing are warranted to validate LAB RNA expression as an alternative biomarker for cancer therapy.

## Figures and Tables

**Table 1:** Univariate and multivariate analysis of major prognostic factors for overall survival using Cox proportional hazards models in cohort 2. Variables significant in univariate analyses were included in multivariate analysis and further selected by backward method.

	Univariate			Multivariate		
	HR	(95% CI)	*p*-Value	HR	(95% CI)	*p*-Value
IHC Score of LAB (1+): LAB > 5% vs. LAB ≤ 5%	3.55	2.30–5.40	<0.0001	5.9	2.61–13.34	<0.0001
Age (in years old): ≥65 vs. <65	1.55	1.06–2.27	0.02	1.64	1.06–2.53	0.028
Smoke: No vs. Yes	1.52	1.02–2.28	0.039	1.27	0.76–2.77	0.36
COPD: Yes vs. No	1.41	0.94–2.21	0.11	1.48	0.87–2.52	0.15
Histology: Squamous vs. Adenocarcinoma	0.87	0.56–1.33	0.51	0.89	0.56–1.41	0.64
Differentiation Grade: Poor (III) vs Well (I) ~Moderate (II)	1.19	0.75–1.88	0.42	1.20	0.75–1.90	0.45

## Data Availability

The dataset supporting the conclusions of this article is included within the article.

## Supplementary Materials

The following supporting information can be downloaded at: https://www.mdpi.com/article/10.3390/cancers15030924/s1. Table S1: The demographics and tumor characteristics for cohort 1 NSCLC cases. Table S2: The demographics and tumor characteristics for cohort 2 NSCLC cases. Table S3: Patient characteristics stratified by expression level of Lab in cohort 2. All statistical tests were Fisher’s exact tests for categorical variables, and Kruskal–Wallis Test for continuous variable. Table S4: Propensity score-weighted survival analysis in cohort 2 for overall survival using the Cox proportional hazards models. Table S5: Univariate survival analysis for overall survival by different LAB expression cutoffs using the Cox proportional hazards models. Table S6: Sensitivity analysis further adjusting for metastasis in multivariate analysis for overall survival using the Cox proportional hazards models in cohort 2. The analysis was based on multiple imputations for unknown metastasis status. Figure S1: LAB IHC Expression in TMAs of LUAD and LUSC.
