# Supplementary material for: Labyrinthin Expression Is Associated with Poor Prognosis in Patients with Non-Small-Cell Lung Cancer
Source: Cancers (Basel). 2023 Feb 1;15(3):924. doi: 10.3390/cancers15030924 (PMC9913764; doi:10.3390/cancers15030924)

Supplemental Data (6 Tables and 1 Figure)

Table S1: Cohort 1

	LUAD (N=37)			LUSC (N=36)			NSCLC Brain Metastasis (N=22)
	Tumor	Normal	p-value[a]	Tumor	Normal	p-value[a]	Tumor
Labyrinthin TPS (%)							
Mean (SD)	36.2 (27.9)	4.4 (6.8)	<0.001	39.1 (31.5)	8.1 (6.2)	<0.001	41.3 (29.1)
Median (range)	25 (0-90)	1 (0-30)		37 (1-92)	6 (0-20)		42.5 (1-95)
Labyrinthin							
0 (0-5%)	3 (8.1%)	30 (81.1%)		8 (22.2%)	16 (44.4%)		1 (4.5%)
1+ (6-20%)	7 (18.9%)	6 (16.2%)	-	4 (11.1%)	18 (50.0%)	-	5 (22.7%)
2+ (21-50%)	14 (37.9%)	1 (2.7%)		13 (36.1%)	2 (5.6%)		8 (36.4%)
3+ (51-100%)	13 (35.1%)	0 (0.0%)		11 (30.6%)	0 (0.0%)		8 (36.4%)
Grade							
Well differentiated	9 (27.3%)			3 (8.6%)			
Moderate differentiated	17 (51.5%)	-	-	20 (57.1%)	-	-	-
Poorly Differentiated	7 (21.2%)			12 (34.3%)			
Age (years old)							
Mean (SD)	63.4 (9.2)			70.6 (8.5)			62.5 (8.3)
Median (range)	64 (47-85)			70.5 (55-95)			63.5 (50-81)
Gender							
Female	23 (62.2%)			13 (36.1%)			11 (50.0%)
Male	14 (37.8%)			23 (63.9%)			11 (50.0%)
Race							
Non-white	12 (32.4%)			28 (77.8%)			11 (50.0%)
White	25 (67.6%)			8 (22.2%)			11 (50.0%)

Abbreviations: LUAD, lung adenocarcinoma; LUSC, lung squamous cell carcinoma; NSCLC, non-small cell lung cancer

Table S2. Cohort 2

Characteristics	N=161	Percentage (%)
Age: (mean ± SD; years old)	65.9 ±10	
Range (years old)	32 - 86	
Gender: N (%)		
Female	94	58.3%
Male	67	41.6%
Race/Ethnicity:		
NHW	121	75.1%
Black	7	4.3%
Asian	4	2.4%
Other	29	18%
Smoking history:		
Smoker	100	62.1%
Never smoker	61	37.9%
Histology type:		
LUAD	91	56.5%
LUSC	46	28.6%
Others	24	14.9%
Grade:		
I	33	20.5%
II	43	26.7%
III	51	31.7%
Not known	34	21.11%

Table S3. NSCLC Patient Characteristics stratified by expression level of Lab in Cohort 2

	IHC≤5% (n=36)	6%≤IHC≤20% (n=39)	21%≤IHC≤50% (n=39)	51%≤IHC≤100% (n=47)	All (n=161)	p value
Age (continuous) Mean (SD)	64.8 (10.5)	66.4 (10.6)	67.0 (8.5)	65.6(10.3)	66.0 (10.0)	0.903
Age (categorical)						0.963
<65	16 (44.4%)	17 (43.6%)	16 (41.0%)	22 (46.8%)	71 (46.8%)	
≥65	20 (55.6%)	22 (56.4%)	23 (59.0%)	25 (53.2%)	90 (53.2%)	
Sex						0.228
Female	25 (69.4%)	24 (61.5%)	18 (46.1%)	27 (57.5%)	94 (58.4%)	
Male	11 (30.6%)	15 (38.5%)	21 (53.9%)	20 (42.5%)	67 (41.6%)	
Race						0.831
Non-white	15 (41.7%)	15 (38.5%)	14 (35.9%)	15 (31.9%)	59 (36.6%)	
White	21 (58.3%)	24 (61.5%)	25 (64.1%)	32 (68.1%)	102 (63.4%)	
Smoking history						0.077
No	8 (22.2%)	13 (33.3%)	18 (46.2%)	22 (46.8%)	61 (37.9%)	
Yes	28 (77.8%)	26 (66.7%)	21 (53.8%)	25 (53.2%)	100 (62.1%)	
COPD						0.981
No	24 (66.7%)	27 (69.2%)	28 (71.8%)	32 (68.1%)	111 (68.9%)	
Yes	12 (33.3%)	12 (30.8%)	11 (28.2%)	15 (31.9%)	50 (31.1%)	
Histology						0.088
Adnocarcino	18 (50.0%)	25 (64.0%)	16 (41.0%)	30 (63.8%)	89 (55.3%)	
Squmaous	10 (27.8%)	7 (18.0%)	18 (46.2%)	13 (27.6%)	48 (29.8%)	
Other/Unknown	8 (22.2%)	7 (18.0%)	5 (12.8%)	4 (8.5%)	24 (14.9%)	
Differentiation Grade						0.014
Well	9 (25.0%)	13 (33.3%)	4 (10.3%)	5 (10.6%)	31 (19.3%)	
Moderate	7 (19.4%)	7 (17.9%)	20 (51.3%)	18 (38.4%)	52 (32.3%)	
Poor	11 (30.6%)	8 (20.5%)	10 (25.6%)	15 (31.9%)	44 (27.3%)	
Unknown	9 (25.0%)	11 (28.2%)	5 (12.8%)	9 (19.1%)	34 (21.1%)	
Metastasis						0.005
No	18 (50.0%)	9 (23.1%)	10 (25.6%)	5 (10.6%)	42 (26.1%)	
Yes	2 (5.6%)	5 (12.8%)	3 (7.7%)	9 (19.2%)	19 (11.8%)	
Unknown	16 (44.4%)	25 (64.1%)	26 (66.7%)	33 (70.2%)	100 (62.1%)	

Table S4: Propensity Score-weighted Survival Analysis in cohort 2 for overall using Cox Proportional Hazards Models.

Lab IHC Score	Propensity Score-Weighted		
	HR	(95% CI)	p-value
**Overall**			
IHC Score of Lab			
LAB ≤5%	1.00		
LAB >5%	2.56	1.88-3.48	<0.001
**Subset Analysis of Smokers**			
IHC Score of Lab			
LAB ≤5%	1.00		
LAB >5%	4.14	2.61-6.57	<0.001
**Subset Analysis of Non-smokers**			
IHC Score of Lab			
LAB ≤5%	1.00		
LAB >5%	1.44	0.94-2.20	0.094
**Subset Analysis of Differentiation**			
Grade I&II			
IHC Score of Lab			
LAB ≤5%	1.00		
LAB >5%	4.35	2.60-7.28	<0.001
**Subset Analysis of Differentiation**			
Grade III			
IHC Score of Lab			
LAB ≤5%	1.00		
LAB >5%	2.91	1.73-4.90	<0.001

Table S5 (cohort 2): Univariate Survival Analysis for Overall Survival by different Lab Expression cut off Using Cox proportional Hazards Models.

Lab IHC Score	Univariate		
	HR	(95% CI)	p-value
IHC Score of Lab (1+)			
Lab≤5%	1.00		
Lab >5%	3.55	2.30-5.40	<0.0001
IHC Score of Lab (2+)			
Lab≤20%	1.00		
Lab >20%	2.05	1.38-3.06	<0.001
IHC Score of Lab (3+)			
Lab≤50%	1.00		
Lab >51%	2.16	1.45-3.23	<0.001

Table S6. Sensitivity analysis further adjusting for metastasis in multivariate analysis for overall survival using the Cox proportional hazards models in cohort 2.

	Multivariate		
	HR	(95% CI)	p-value
IHC Score of LAB (1+)<br>LAB >5% vs LAB ≤5%	3.65	1.57-8.46	0.004
Age (in years old)<br>≥65 vs <65	1.50	0.91-2.49	0.121
Smoke<br>No vs Yes	0.81	0.44-1.52	0.524
COPD<br>Yes vs No	2.00	1.06-3.75	0.036
Histology<br>Squamous vs Adenocarcinoma<br>Other vs Adenocarcinoma	 2.32<br>0.58	 1.21-4.43<br>0.24-1.41	 0.015<br>0.237
Differentiation Grade<br>Poor (III) vs<br>Well (I) ~Moderate (II)	1.15	0.67-1.97	0.616
Metastasis<br>Yes vs No	11.10	4.11-29.99	<0.001

Figure S1. LAB IHC Expression in TMAs

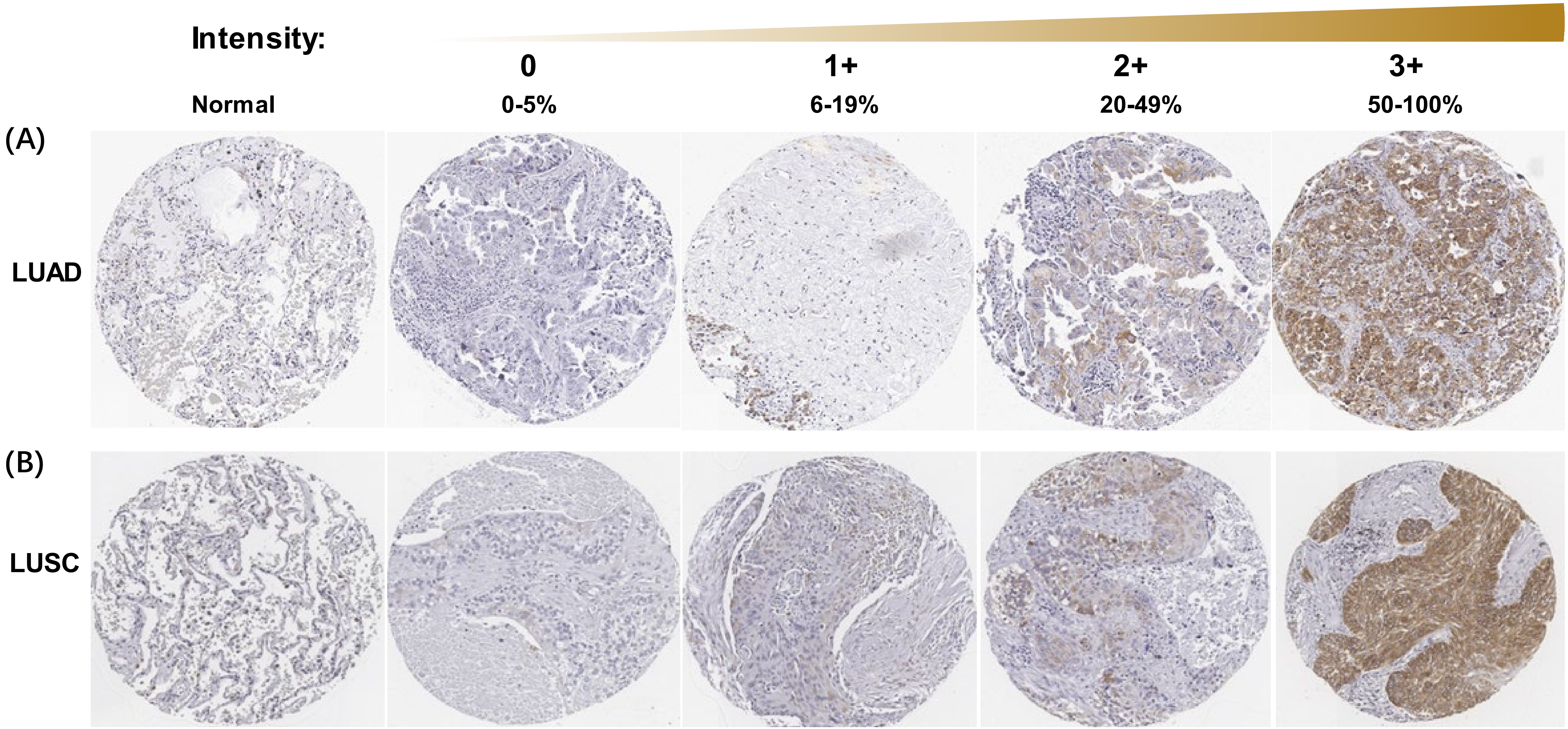

Supplement: Supplementary file 1 [file cancers-15-00924-s001.zip › cancers-2099928-supplementary.pdf]
